# Paper on “Trench Feet” Prevention

**Published:** 1918-12

**Authors:** R. S. H. Fuhr


**Paper on "Trench Feet" Prevention.** By Col. R. S. H. Fuhr, C. M. G., D. S. O., British Army.

The reduction of sick wastage from trench feet is one of the great medical problems during the winter and early spring.

Under the most favorable circumstances, and with skilled treatment, the sufferer is lost to the fighting strength from 14 days to 3 months.

A first attack predisposes to recurrence owing to the thickening and induration of the parts primarily affected, with consequent poor blood supply.

Severe cases, in which loss of toes, etc., has occured, may cause lowering of Category or invaliding from the Service.

The wastage in one army during the winter 1916-1917 was 5822 cases evacuated to the Base out of 6107 admissions.

From the above, the necessity of efficient prophylaxis strictly adhered to, is obvious.

The principles carried out in accordance with the various orders that have been in force on the subject of trench feet in the British Armies during the last few years are designed to counteract predisposing causes, which are :

Wet muddy trenches.

Dirty feet.

Lowering of the circulation in the lower limbs due to constriction of the legs and feet by ill-fitting boots, tight puttees, tight socks shrunk by washing, tight tapes at end of drawers, maintenance of cramped positions for long periods in Forward Posts where any movement, except at night, is impossible, owing to visibility by the enemy.

Strain and lowered resistance due to prolonged exposure.

Lack of hot nourishing food.

It should be borne in mind that the object we all have in view is to defeat the enemy, and that orders concerning trench feet must be compatible with the military situation, and involve as little interference as possible with its demands.

Commanding officers must be reminded that the loss of effective strength due to the prevalence of this trouble is an indication of faulty discipline and faulty interior economy, and they will, therefore, be held responsible that the instructions laid down are carried out under the strict supervision of company officers.

Regular feet rubbing drills will be carried out throughout the army during inclement weather both in and out of the trenches. Such drills improve the circulation and should be started long before the men go into the trenches.

Trench foot is favored by standing in wet boots and is aggravated by the use of tight boots, tight puttees and the use of anything causing constriction of the lower limbs,

Action on the following lines, in addition to the preventive treatment given above, will therefore be carefully and systematically carried out.

*a*) Trenches will be kept as dry as possible.

*b*) Men in water-logged trenches will be relieved every 24 hours if possible.

*c*) Men holding water-logged trenches will wear gum boots. If these are not available, the ankle boot or field service boot worn should be at least two sizes too large and very loosely laced. When gum boots are worn, the socks should be fastened round the ankle by a safety pin, so as to prevent them working down under the heel. No form of garter is to be worn.

*d*) Puttees should not be worn in the trenches, and the tapes attached to certain patterns of drawers to prevent the drawers from rucking up the leg, are not to be tied tightly.

*e*) The preventive treatment will be carried out under the direct personal supervision of an officer.

*f*) A second pair of dry socks will be carried by each man, and battalion arrangements will be made for dry clean socks to be sent up in water-proof bags, daily, with the supplies for the men in the trenches.

*g*) While in the trenches men should be encouraged to move about as much as possible so as to maintain blood circulation. On no account will men be allowed to hold their feet near a fire or dip them into hot water.

*h*) Arrangements will be made whereby men in the trenches can obtain warm food, hot drinks, shelter and warmth. Where conditions are favorable, a Regimental Post will be instituted in proximity to the trenches, where men who show signs of suffering from exposure can be attended to promptly.

*i*) On coming out of the trenches after a tour of duty, every man should remove his wet boots and socks, thoroughly dry rub the feet and put on dry socks and dry boots. Scrupulous care should be taken to see that facilities exist, and that the men carry out these details as soon as they get into reserve billets. As much preventable harm can be done by the men hanging about in billets with sodden boots and socks as may result from hours of exposure in the front trenches.

*j*) Here follow full details for the provision of gum boot stores, the drying and marking of same, and the cleaning, drying, and greasing of ankle boots left in the store.

A further example is in the form of a suitable notice posted in places where it is likely to be read by the men.

PRECAUTIONS TO BE TAKEN BY ALL RANKS AGAINST TRENCH FOOT AND FROST BITE.

1. *Before starting for the trenches.*

*a*) *Wash feet* in water with the chill just off. Water must not be hot or it will do more harm than good.

*b*) *Dry feet* thoroughly.

*c*) *Rub feet* well with about one ounce of whale oil.

*d*) *Put on dry socks* which must not be tight. If tight ask for another pair.

*e*) *Lace your boots very loosely* and put your puttees on quite loosely.

2. *When you start off see that you have* —

*a*) At least one pair of *dry* socks.

*b*) Some spare whale oil.

*c*) A towel.

3. *In trenches.*

*a*) When possible, take your boots off once a day.

*b*) Get someone to rub your feet with whale oil while you rub his.

*c*) Then put on *dry* socks.

4. *When you arrive back from the trenches and before you go to sleep* —

*a*) Wash feet in water with chill just taken off. Do not wash in warm water.

*b*) Dry feet well.

*c*) Get someone to rub your feet for some minutes and you rub someone else's feet.

*d*) Put on a pair of *dry* socks — if you have none, ask your Platoon Sergeant.

Whatever the method of prophylaxis adopted and thoroughly carried out, the determining factor, military necessity versus sick wastage, must be settled by the General Staff. The duration of the exposure in Forward Posts, etc., depends on their order, and with them lies the ultimate responsibility.

*Failure of Prophylaxis.*

The causes of failure may also be due to —

*a*) Climatic conditions, such as —

Excessive rain filling trench thigh deep with mud.

Frost followed by thaw with rain producing icy cold liquid mud.

*b*) Low lying marshy ground well under observation by the enemy, which prevents movement by day and makes reliefs costly in casualties.

*c*) Strain of holding a large front with a weak and tired division.

*d*) Dirty feet.

*e*) Bad discipline and interior economy.

*f*) Lack of co-operation for the common good between the staff and departments concerned.

*Prophylactic Measures* may be summarized briefly as follows :

1. Cleanliness and care of the feet.
2. Maintenance of good discipline and personal supervision by officers.
3. Provision of hot food, dry socks, etc., in the trenches.
4. Limitation of time of exposure.
5. Improvement in local conditions.
6. Stamping exercises to promote circulation.
7. Rigid carrying out by all ranks of the particular measures decided upon.

*Cleanliness and Care of the Feet.*

Whale oil as a local application to the feet was used for a long time, but it is dirty, cold and clammy when applied, and has no specific action.

The result of a thorough trial, in one division, of the French treatment for " Trench Foot ", lasting from 6-2-17 to 21-3-17, proved this treatment to be of the greatest value in saving wastage from a preventable disease and its permanent adoption is strongly recommended.

This method of prophylaxis consists of :

1. *Cleanliness of the feet* by washing with special soap.
2. *Powdering the socks* with a medicated powder.
3. *Careful attention to the fitting of the boots*, thorough upkeep of same, with regular application of dubbin.
4. *The usual stamping exercises and plenty of work.*
5. *Supply of hot tea in the trenches.*

The object of these measures is to keep the men's feet clean and dry, to stimulate the circulation, and keep up the general well-being of the men.

*The soap used is composed of* :

| | |
|---|---|
| Soft potash soap . . . . . . . . . . . . . . . | 1000 parts. |
| Powdered camphor . . . . . . . . . . . . | 25 " |
| Powdered borax . . . . . . . . . . . . . . . | 100 " |

It is made in " Soyers " stoves by heating the potash soap until it is liquid, but not boiling, stirring in the camphor until well dissolved, stirring in the borax likewise, then removing from the fire and keeping on stirring until set.

*The powder is made up of*:

| | |
|---|---|
| Talc . . . . . . . . . . . . . . . . . . . . . . | 1000 parts. |
| Camphor . . . . . . . . . . . . . . . . . . . . | 25 " |

The amount of soap required for each foot wash per man is 1/4 of an oz., or roughly 3/4 of a petrol tin full for a battalion.

The amount of powder required is 10 lbs., or half a petrol tin full for a battalion (640 strong).

The soldier's feet are washed, dried, and clean powdered socks are put on before he goes into the trenches. While in the front line, socks are changed daily, or taken off, feet wiped dry, fresh powder dusted on, and a thorough rubbing and massage carried out.

In wet weather the initial foot bath is to be repeated in 3 or 4 days.

The above mentioned period, during which this method was tried, comprised hard frost for the first 13 days, followed by a severe thaw.

The trenches, when the thaw set in, were nearly knee deep, in many places, with icy slush over tenacious cold mud.

123 cases of trench foot were noted, and 17 of these were relapses in men who had comparatively recently been discharged from hospital as cured of trench foot.

While the care and attention to the cleanliness of the feet is of great value, it is believed that camphor has a specific action on the casual organism, and acts also as a stimulant to the circulation of the feet.

The washing and powdering are very popular with the men, who are convinced of their efficacy.

*Table of Cases.*

| Admissions. | To C. C. S. | To duty. | Remaining. | Disease changed. |
|---|---|---|---|---|
| 123 | 42 | 51 | 13 | 17 |

The above table shows the incidence and disposal of cases of this disease.

The total strength of men in the Forward Area at any given time, to which the above figures refer, was about 6000.

*Comparative Table.*

| Whale oil treatment period. | | French treatment period. | |
|---|---|---|---|
| Admissions . . . . . . | 529 | Admissions . . . . . . | 123 |
| Percentage of cases . . | 8.8 | Percentage of cases . . | 2.05 |

In making the above comparison, it should be borne in mind that the French prophylactic treatment was tried when the division had already suffered from severe exposure.

